# Acacetin inhibits expression of matrix metalloproteinases *via* a MAPK-dependent mechanism in fibroblast-like synoviocytes

**DOI:** 10.1111/jcmm.12564

**Published:** 2015-04-09

**Authors:** Wei-Ping Chen, Zhi-Gao Yang, Peng-Fei Hu, Jia-Peng Bao, Li-Dong Wu

**Affiliations:** Department of Orthopedic Surgery, The Second Affiliated Hospital, School of Medicine, Zhejiang UniversityHangzhou, Zhejiang Province, China

**Keywords:** acacetin, rheumatoid arthritis, matrix metalloproteinase, fibroblast-like synoviocytes, interleukin-1β

## Abstract

It is well known that rheumatoid arthritis (RA) is an autoimmune joint disease in which fibroblast-like synoviocytes (FLSs) play a pivotal role. In this study, we investigated the anti-arthritic properties of acacetin in FLSs. The expression of matrix metalloproteinase (MMP)-1, MMP-3 and MMP-13 were investigated by quantitative RT-PCR and western blot at gene and protein levels. At the same time, the phosphorylation of mitogen-activated protein kinases (MAPK) was investigated. The DNA-binding activity of NF-κB was investigated by electrophoretic mobility shift assay. We found that *acacetin* inhibits p38 and JNK phosphorylation and reduces MMP-1, MMP-3 and MMP-13 expression in interleukin-1β-induced FLSs. Our results suggest that acacetin has antiarthritic effects in FLSs. Thus, acacetin should be further studied for the treatment of arthritis.

## Introduction

Rheumatoid arthritis (RA) is a chronic autoimmune disease involving proliferative synovitis and cartilage destruction at joints. Although its aetiology remains elusive, fibroblast-like synoviocytes (FLSs) are known to play a pivotal role in RA pathophysiology 1. The activation of FLSs results in over-production of pro-inflammatory cytokines, including tumour necrosis factor (TNF)-α and interleukin (IL)-1β, which are major factors in RA pathophysiology 2. These cytokines induce production of inflammatory mediators and matrix metalloproteinases (MMPs) 3,4, and cause activation of other inflammatory cells such as T cells, all of which contribute to the progression of RA 5. Activated FLSs also produce MMPs 6, which are the matrix-degrading enzymes responsible for extracellular matrix destruction and cartilage degradation 7.

Anti-inflammatory treatment is currently an important intervention for patients with RA. The ameliorative effects of non-steroidal anti-inflammatory drugs and selective cyclooxygenase (COX)-2 inhibitors are associated with their anti-inflammatory properties. However, side effects in the gastrointestinal and cardiovascular systems limit long-term use of these pharmacological treatments 8,9. Similarly, anti-TNF-α therapy, although effective for RA, also causes hypersensitivity to medications and increased likelihood of infection 10,11. Thus, safer effective anti-inflammatory agents are needed for the treatment of RA.

Flavonoids are contained in fruits, vegetables, and other components of the human diet. Previous studies have reported beneficial antitumour and antioxidative actions of flavonoids 12–14. The flavonoid acacetin (5,7-dihydroxy-4′-methoxyflavone) is known to possess anti-peroxidative and anti-cancer properties against various cancerous cell lines 15–17. Recent studies have demonstrated that acacetin also exerts anti-inflammatory effects in cells 18. Acacetin was reported to inhibit the expression of COX-2, inducible nitric oxide synthase and prostaglandin E2 as well as nitrite production in lipopolysaccharide-induced RAW 264.7 cells 19, and to reduce MMP-2 and MMP-9 expression in several cell lines 20,21. Its inhibitory influence on inflammatory responses and MMPs suggests that acacetin may be beneficial for treating inflammatory diseases such as RA. Despite the importance of pro-inflammatory cytokines and MMPs in RA, little is known about the effects of acacetin on FLSs in experimental RA. This study investigated the anti-inflammatory effects of acacetin in FLSs, including its effects on MMPs in FLSs induced by IL-1β.

## Materials and methods

### Reagents

Acacetin, recombinant human IL-1β, collagenase and 3-(4-5-dimethylthiazolyl-2)-2-5-diphe-nyltetrazoliumbromide (MTT) were purchased from Sigma-Aldrich (St. Louis, MO, USA). DMEM, RPMI 1640, penicillin, streptomycin, foetal bovine serum and 0.25% trypsin were obtained from Gibco-BRL (Grand Island, NY, USA).

### Culture of FLSs

The study was approved by the Local Ethics Committee. Following patient consent, synovial tissues were obtained from RA patients undergoing total knee arthroplasty. Patients had been diagnosed based on American College of Rheumatology clinical criteria. FLSs were isolated as previously described 22. In brief, FLSs were obtained by digesting synovial tissues in RPMI 1640 supplemented with 1 mg/ml collagenase (Gibco-BRL) at 37°C for 90 min. The cells were cultured with DMEM supplemented with 10% foetal bovine serum and antibiotics (100 U/ml penicillin and 100 mg/ml streptomycin) at 37°C in a humidified atmosphere of 5% CO_2_/95% air. Confluent cells were passaged at a 1:3 ratio, and FLSs were used after 3–5 passages.

### MTT assay

Cells were seeded in a 96-well plate (6 × 10^3^/well) and cultured with various concentrations of acacetin for 24 hrs. Then, 20 μl of MTT (5 mg/ml) were added, and incubation was continued for 4 hrs. The culture medium was removed, 150 μl of dimethyl sulphoxide were added, and the absorbance was measured at 570 nm using a micorplate reader (Bio-Rad, Hercules, CA, USA).

### Cell treatments

FLSs (1 × 105/well in six-well plates) were starved in serum-free medium overnight. Some of the cells were treated with acacetin for 1 hr, followed by stimulation with IL-1β (10 ng/ml) for 24 hrs. The cells were harvested for quantitative real-time PCR analysis and western blot analysis to investigate MMPs *gene and protein* expression. Other cells were pre-treated with acacetin for 2 hrs, stimulated with IL-1β (10 ng/ml) for 30 min., and then harvested for western blotting to investigate the effects of acacetin on mitogen-activated protein kinase (MAPK) signalling pathways.

### ELISA

Fibroblast-like synoviocytes were starved in serum-free medium overnight. Some of the cells were treated with acacetin for 1 hr, followed by stimulation with IL-1β (10 ng/ml) for 24 hrs. Conditioned media was collected for IL-6 measurement with an ELISA kit (R&D Systems, Minneapolis, MN, USA).

### Quantitative RT-PCR analysis

Total RNA was extracted using TRIzol reagent (Invitrogen, Carlsbad, CA, USA) according to the manufacturer’s instructions, and 2 μg was reverse transcribed using 1 μl of oligo(dT)_18_ primer, 25 units of RNase inhibitor, 2 μl of dNTPs (10 mM) and a Moloney Murine Leukemia Virus reverse transcriptase cDNA synthesis kit (Promega, Madison, WI, USA). Quantitative real-time PCR was performed with an iQTM SYBR Green Supermix PCR kit with an iCycler system (Bio-Rad, Hercules, CA, USA) under conditions of 95°C for 10 min., followed by 40 cycles at 95°C for 15 sec. and 60°C for 60 sec. The primers used are shown in Table1. GAPDH was amplified as an internal control. The RT-PCR data were quantified using the 2^−ΔΔCt^ method.

**Table 1:** Primers of targeted genes

Targeted genes	Sequence (5′–3′)	Amplicon length (bp)	Accession number
MMP-1	S: GGGAATAAGTACTGGGCTGTTCAG	125	NM_002421
	A: CCTCAGAAAGAGCAGCATCGATATG		
MMP-3	S: CCAATCCTACTGTTGCTGTGCGT	91	J03209
	A: CTGAACAAGGTTCATGCTGGTGTC		
MMP-13	S: CTGGCCTGCTGGCTCATGCTT	162	NM_002427
	A: GCAGGGTCCTTGGAGTGGTCA		
GAPDH	S: CTGCTCCTCCTGTTCGACAGT	100	NM_002046
	A: CCGTTGACTCCGACCTTCAC		

* S: Sense; A: Antisense.

### Western blot analysis

Cells were washed twice with ice-cold phosphate-buffered saline, harvested by scraping and centrifugation, and treated with lysis buffer containing 50 mM Tris-Cl, pH 7.4, 150 mM NaCl, 1 mM EDTA, 1 mM EGTA, 10 μg/ml aprotinin, 10 μg/ml leupeptin, 5 mM phenylmethylsulfonyl fluoride, 1 mM DTT and 1% Triton X-100 for 30 min. on ice. The lysates were centrifuged at The lysates were centrifuged. for 15 min. at 4°C. Lysate proteins were resolved by SDS-PAGE and transferred to Polyvinylidene Fluoride membranes. The membranes were incubated in blocking buffer consisting of skimmed milk powder (5% w/v) and 0.1% Tween 20 in 1× Tris-buffered saline for 1 hr and then incubated with rabbit polyclonal antibodies against MMP-1, MMP-3, MMP-13, p-ERK1/2, p-P38, p-Jun N-terminal kinase (JNK) or β-actin (Cell Signaling Technology, Beverly, MA, USA) overnight at 4°C. The membranes were then washed and incubated for 1 hr at room temperature with goat anti-rabbit secondary antibody. Immunoreactive bands were detected by enhanced chemiluminescence and exposure to X-ray film (Kodak, Hangzhou, China).

### Electrophoretic mobility shift assay

Cells (1 × 10^6^ cells) were seeded in 100-mm dishes and serum-starved overnight. Then, the cells were treated with acacetin for 30 min. and stimulated with IL-1β (10 ng/ml) for 90 min., after which the cells were washed in ice-cold phosphate-buffered saline and collected by scraping. *Nucleoprotein* extraction was performed with a Nucleus Protein Extraction Kit (Viagene Biotech, Ningbo, China). 2 μl nuclear proteins were mixed with 1.5 μl 10× binding buffer, 1.0 μl poly (dI:dC), 0.5 μl biotin-labelled nuclear factor (NF)-κB probe and ddH_2_O in a 15-μl reaction volume and were incubated for 20 min. at room temperature. Polyacrylamide gel electrophoresis was performed at 180 V for 70 min., and the proteins were transferred to the binding membrane in 0.5× TBE at 390 mA for 40 min. To crosslink the DNA, the membrane was exposed to UV light for 5 min. and then blocked. The bands were visualized by chemiluminescence reaction. Images were obtained using the Cool II Imager System (Viagene Biotech).

### Statistical analysis

Data are expressed as means ± SD. Statistical significance was assessed by a paired Student’s *t*-test. Differences were considered significant for *P* < 0.05.

## Results

### Effect of acacetin on viability of FLSs

**Figure 1:**
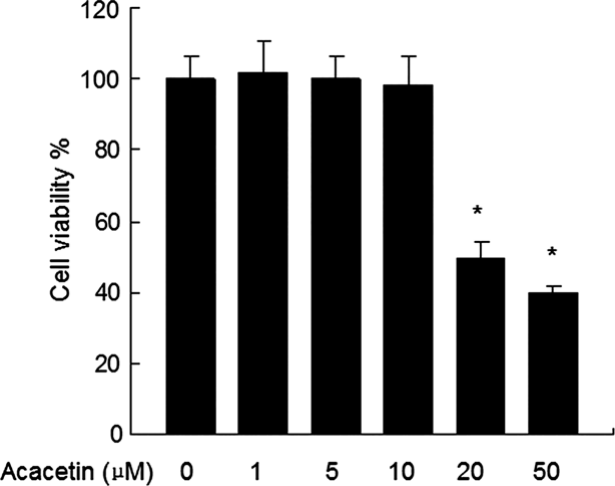
Effect of acacetin on cell *viability*. Cells were seeded in 96-well plates at 6 × 10^3^/well and cultured with different concentrations of acacetin for 24 hrs, followed by MTT assay analysis. Data are expressed as means ± SD. *P < 0.05 compared with cells treated with culture medium only.

MTT assays showed that acacetin at the concentrations of 1, 5 and 10 μM showed no cytotoxicity. However, higher concentrations showed significant cytotoxicity (Fig.1). Thus, 10 μM acacetin was used in subsequent experiments.

### Effect of acacein on IL-6 production

**Figure 2:**
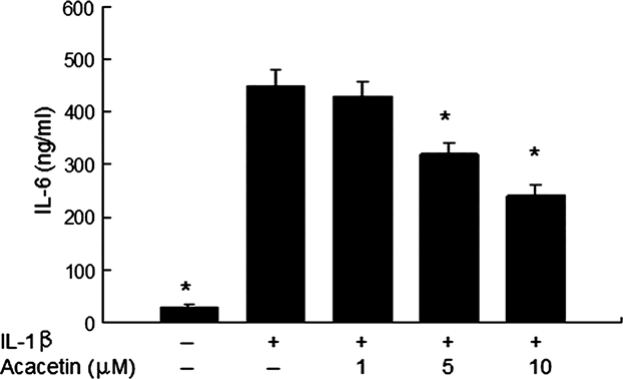
Effect of acacetin on IL-6 production. Cells (1 × 10^5^/well in six-well plates) were treated with acacetin for 1 hr prior to treatment with IL-1β (10 ng/ml) and were collected after 24 hrs. Conditioned media was collected for IL-6 measurement with an ELISA kit. Data are expressed as means ± SD. *P < 0.05 compared with cells stimulated with IL-1β in the absence of acacetin.

The production of IL-6 in conditioned media was detected by ELISA. IL-1β stimulation significantly increased the production of IL-6 and acacetin inhibited IL-6 in a dose-dependent manner (Fig.2).

### Effect of acacetin on the expression of MMP-1, MMP-3 and MMP-13 in FLSs

**Figure 3:**
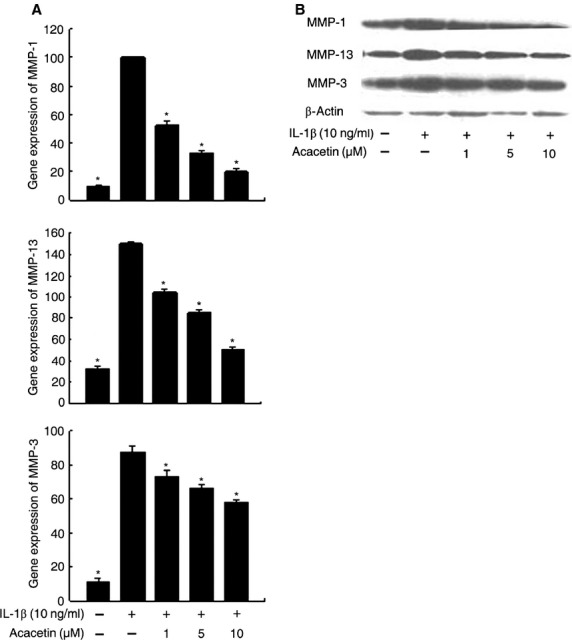
Effect of acacetin on MMP-1, MMP-3 and MMP-13 expression in FLSs. Cells (1 × 10^5^/well in six-well plates) were treated with acacetin for 1 hr prior to treatment with IL-1β (10 ng/ml) and were collected after 24 hrs. (A) Quantitative real-time PCR and (B) Western blot analyses were performed to analyse MMP-1, MMP-3 and MMP-13 mRNA and protein expression respectively. *P < 0.05 compared with cells stimulated with IL-1β in the absence of acacetin.

The induction of MMP-1, MMP-3 and MMP-13 gene expressions in IL-1β-stimulated FLSs was assessed by quantitative real-time PCR. Acacetin inhibited MMP-1, MMP-3 and MMP-13 gene expressions in a dose-dependent manner (Fig.3A). MMP-1, MMP-3 and MMP-13 protein expressions were also affected (Fig.3B).

### Effects of acacetin on IL-1β-induced MAPK signalling pathways

**Figure 4:**
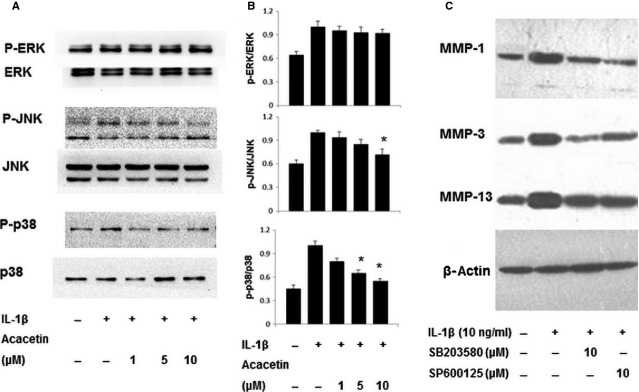
Effect of acacetin on IL-1β-induced phosphorylation of MAPKs in FLSs. Western blotting was performed to detect the phosphorylation levels of JNK, ERK1/2 and p38 in cells pre-treated with acacetin for 2 hrs before stimulation with IL-1β for 30 min. (A). The ratio between the intensity of the phosphorylated and non-phosphorylated band are shown in B. Cells were treated with a p38 inhibitor or a JNK inhibitor for 2 hrs before stimulation with IL-1β for 30 min., and MMP-1, MMP-3 and MMP-13 expression *were* assessed (C). *P < 0.05 compared with cells stimulated with IL-1β in the absence of acacetin.

As MAPK pathways are involved in the regulation of MMP expression as well as the inflammatory process, the effects of acacetin on MAPKs were investigated. IL-1β significantly induced the phosphorylation of ERK1/2, p38 and JNK within 30 min. The phosphorylation of p38 and JNK was inhibited by acacetin, whereas the phosphorylation of ERK1/2 was not affected (Fig.4A). The ratio between the intensity of the phosphorylated and non-phosphorylated band are shown in Figure4B. Furthermore, pre-treatment of FLSs with either a p38 inhibitor (SB203580; 10 μM) or a JNK inhibitor (SP600125, 10 μM) for 2 hrs before stimulation with IL-1β for 30 min. reduced IL-1β-induced protein expressions of MMP-1, MMP-3 and MMP-13 (Fig.4C).

### Effects of acacetin on NF-κB activation

**Figure 5:**
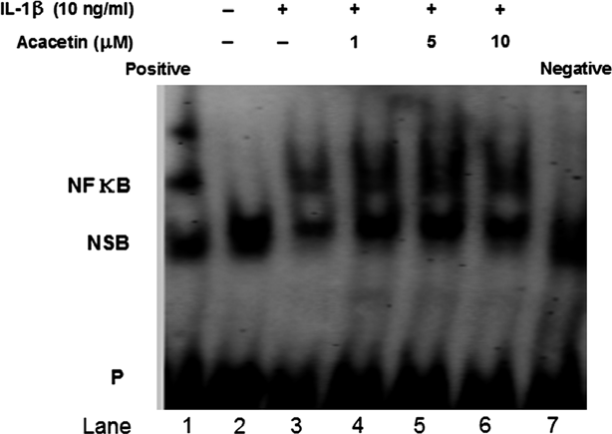
Effect of acacetin on DNA-binding activity of NF-κB. Cells were pre-treated with various concentration of acacetin for 30 min. before stimulation with IL-1β (10 ng/ml) for 90 min. Nuclear extracts were prepared and analysed by electrophoretic mobility shift assay. Lane 1: nuclear extracts incubated with unlabelled consensus oligonucleotide (Positive) to confirm the specificity of binding. Lane 2–6 biotin-NF-κB oligonucleotide plus IL-1β and various concentrations of acacetin. Lane 7 biotin-NF-κB oligonucleotide (Negative), no nuclear extracts for probe to bind. IL-1β stimulation increased nuclear levels of NF-κB, and acacetin did not inhibit the DNA-binding activity of NF-κB. NSB: Non-Specific Binding; P: Free Biotin-labelled Probe.

Although the NF-κB signalling pathway is also important in regulating MMPs and inflammatory factors, electrophoretic mobility shift assay results showed that acacetin did not inhibit the DNA-binding activity of NF-κB (Fig.5).

## Discussion

The results of this study demonstrate that acacetin inhibits p38 and JNK phosphorylation and reduces MMP-1, MMP-3 and MMP-13 expression in IL-1β-induced FLSs.

The role of pro-inflammatory cytokines in RA has been well-established 23. IL-1β is the predominant pro-inflammatory cytokine involved in joint destruction associated with RA 24. In the light of its catabolic effects, IL-1β has been widely used to mimic arthritis in *in vitro* studies 25. In the present study, IL-1β significantly induced the expressions of MMP-1, MMP-3 and MMP-13 in FLSs. In addition, acacetin inhibited the IL-1β-induced gene and protein expressions of MMP-1, MMP-3 and MMP-13 in FLSs. Given that MMP-1, MMP-3 and MMP-13 play pivotal roles in the cartilage matrix breakdown associated with both RA and osteoarthritis, the inhibitory effects of acacetin on MMPs indicates a potential role for acacetin in preventing cartilage degradation associated with RA.

Mitogen-activated protein kinases, including JNK, ERK and p38, are involved in the regulation of MMP 26, suggesting that MAPK pathways may be a molecular mechanism for acacetin inhibition of MMPs in IL-1β-induced FLSs. In this study, IL-1β stimulation of FLSs resulted in the phosphorylation of MAPKs, confirming the role of IL-1β in RA. Furthermore, 10 μM acacetin inhibited the phosphorylation of p38 and JNK, but not the phosphorylation of ERK. A p38 inhibitor and a JNK inhibitor significantly decreased the protein expressions of MMP-1, MMP-3 and MMP-13 in response to IL-1β. These findings suggest that acacetin inhibits the p38 and JNK signalling pathways to reduce MMPs expression. The inhibition of MAPK pathways by acacetin has been previously described. Shen *et al*. 21 have shown an inhibitory effect of acacetin on the phosphorylation of p38 in DU145 cells. Interestingly, Fong *et al*. 20 reported the inactivation of JNK signalling in A549 cells treated with acacetin, but p38 and ERK were not inhibited by acacetin. This apparent discrepancy may be attributable to cell type or stimulation conditions. Moreover, more than 20 of p38MAPK inhibitors were found by various pharmaceutical companies and none of them had therapeutic effects on RA, the reason why acacetin could exert anti-inflammatory/anti-arthritic effects may be by inhibiting not only the activation of p38 *MAPK* signalling pathway, but also JNK signalling pathway.

It is well known that the NF-κB pathway is important in the pathogenesis of chronic inflammatory diseases 27. The activation of NF-κB results in the up-regulation of inflammatory genes 28 and the induction of MMPs in arthritis 29. In the present study, the IL-1β induced DNA-binding activity of NF-κB was not affected by acacetin. However, acacetin did affect NF-κB-dependent gene expressions downstream of DNA-binding in FLSs. The current findings are not in accordance with previous studies showing that acacetin suppresses DNA-binding of NF-κB in DU145 cells and in 12-O-tetradecanoylphorbol-13-acetate-induced A549 cells 20,21. This disparity may be attributable to differences in acacetin exposure time or cell type.

In conclusion, we demonstrated that acacetin inhibited the expression of MMP-1, MMP-3 and MMP-13 at gene and protein levels in IL-1β-stimulated FLSs. The inhibitory effect is associated partly with inhibition of the p38 and JNK pathways and independent of the DNA-binding activity of NF-κB.
